# Gemcitabine in combination with EGF-Receptor antibody (Cetuximab) as a treatment of cholangiocarcinoma: a case report

**DOI:** 10.1186/1471-2407-6-190

**Published:** 2006-07-17

**Authors:** Martin F Sprinzl, Carl C Schimanski, Markus Moehler, Simin Schadmand-Fischer, Peter R Galle, Stephan Kanzler

**Affiliations:** 1 I. Department of Medicine, Johannes-Gutenberg University, Mainz, Langenbeckstrasse 1, 55101 Mainz, Germany; 2 Department of Radiology, Johannes-Gutenberg University, Mainz, Langenbeckstrasse 1, 55101 Mainz, Germany

## Abstract

**Background:**

Extensive disease of cholangiocarcinoma (CC) determines the overall outcome and limits curative resection. Despite chemotherapy, which has been introduced to improve the outcome of biliary tract malignancies, the benefit in survival is still marginal.

**Case presentation:**

We report a 69-year-old patient with non-resectable CC showing hepatic metastasis and peritoneal carcinomatosis. Diagnosis was based on computed tomography, mini-laparoscopy and bioptic specimens. Histology revealed an adenocarcinoma of the biliary tract with expression of epithelial growth factor receptor. After informed consent the patient received experimental gemcitabine (1000 mg/m^2^) every other week and cetuximab (250 mg/m^2^) weekly for palliative chemotherapy. During the reported follow up (since time of first presentation) 20 cycles of chemotherapy were administered. Relevant chemotherapy-related toxicity was limited on gemcitabine-associated side effects. Predominantly, haematological toxicity (CTC, grade 3) and neutropenic fever (CTC, grade 3) promoted by catheter-related sepsis were observed. Cetuximab caused only mild skin toxicity (CTC, grade 1).

Chemotherapy led to a partial response (> 30% reduction, according to RECIST) of the target lesions and disappearance of the peritoneal carcinomatosis as shown by computed tomography. Partial response occurred after 17 weeks of treatment and remained stable during the entire course of chemotherapy for 9.7 months. In parallel, Ca 19-9 serum levels, which were elevated 5-fold at time of diagnosis, returned to normal after 16 weeks of treatment. The performance status stabilized and intravenous alimentation could be discontinued.

**Conclusion:**

Our experience from one patient with CC suggests, that a combination of cytotoxic chemotherapy together with cetuximab may show promising efficacy in respect to survival and quality of life. Therefore cetuximab, as a component of palliative chemotherapy in biliary tract cancer, needs further evaluation in prospective randomized trials.

## Background

Most patients suffering from cholangiocarcinoma (CC) show extensive local disease hampering curative resection. After resection local recurrence and metastatic disease often determines the overall outcome. Therefore, treatment of these patients with potent cytotoxic chemotherapies is a crucial issue. In addition, patients with resectable tumors have a high recurrence rate with a 5-year survival rate of 9–18% for proximal peri-hepatic lesions and 20–30% for more distal malignant manifestations [1]. Adjuvant therapy did not improve survival rates in context of biliary tract cancer and is therefore not advised. The only entity of biliary tract cancer, which may profit from adjuvant chemotherapy, might be gall bladder cancer [2,3]. Chemotherapy has been commonly used to improve outcome and to control tumor progression. Different chemotherapeutic agents have been employed. The results are yet limited on improved quality of life with reduced analgesic requirement during supportive care. Prolongation of median survival in comparison to best supportive care remained marginal (6 months vs. 2.5 months) during palliative chemotherapy [4]. Gemcitabine is a promising agent, which has shown efficacy in biliary tract cancer. Gemcitabine is administered as single agent or in combination with other cytotoxic partners. The response rates on single agent gemcitabine range from 8 to 60 percent, depending on the cohort reported [5]. The average study populations are limited in size with the largest phase II study reporting results from 39 patients. New targeted therapies directed against epithelial growth factor receptor (EGFR), which is frequently overexpressed on transformed cells, have improved outcome in other malignant entities, predominantly colorectal cancer. As a recent phase II study indicates, that EGFR-targeted therapy in combination with gemcitabine might improve the outcome in pancreatic cancer [6], we initiated an experimental course of gemcitabine in combination with EGFR antibody (cetuximab) for a patient suffering from unresectable CC.

## Case presentation

### Clinical presentation

A 69 Year old male presented to our clinic with abdominal discomfort, nausea and a weight loss of 18 kg within four months. The overall performance status was significantly reduced (Karnofsky-Index: 70%). A palpable spleen and tenderness of the right lower abdominal quadrant were found during physical examination, whereas no other relevant pathological findings were evident. Initial computed tomography of the abdomen and thorax revealed a hepatic mass and was indicative for peritoneal carcinomatosis. The tumor was located adjacent to the gallbladder and administration of oral contrast medium before computed tomography did not show any luminal tumor of the intestine. Without evidence for intra or extra-hepatic cholestasis, laboratory findings during first clinical presentation showed inflammatory changes (c-reactive protein: 345 mg/l, normal range < 5 mg/l) and elevated γ-glutamyl transpeptidase serum-levels (5-fold). These findings were suggestive for tumor-associated cholangitis and were treated with wide spectrum intravenous antibiotics (piperacillin/tazobactam).

Subsequent diagnostic mini-laparoscopy as well as hepatic and peritoneal biopsy confirmed the diagnosis of a CC and proved associated peritoneal carcinomatosis. Therefore, no curative resection was applicable due to extensive tumor disease. Histology from the peritoneal and hepatic biopsy showed an intermediately differentiated adenocarcinoma. As the immune-histochemical staining has been negative for CK20 and intensively positive for CK7, a carcinoma from gastrointestinal origin was excluded [7,8]. Spin cytology from aspirated ascites showed coherent findings. Finally, a subsequent analysis for EGFR expression confirmed a weak (1+) tissue staining. After histological diagnosis, the initiation of chemotherapy was delayed, as the subsequent clinical course was complicated by obstipation and protracted bowel obstruction-like symptoms, which finally resolved under laxative therapy.

### Past medical history

Medical history was remarkable for an insulin dependant diabetes mellitus (type 2) for twenty years, associated diabetic nephropathy and polyneuropathy. Adipositas (BMI: 34.8) and hyperuricaemia completed the pre-existing metabolic syndrome. Essential arterial hypertension and absolute arrhythmia, secondary to atrial flutter, showed an uncomplicated clinical course under medical treatment. The patient received amlodipine, ramiprile and torasemide as antihypertensive treatment. Digitoxin and phenprocoumon was administered for heart rate control and embolic prophylaxis, respectively. Facing the extensive CC, phenprocoumon was discontinued and switched to a therapeutical dose of fractionated low molecular heparin. Insulin (10 IU/day), pravastatin and allopurinol were further co-medications at time of first admission. An analgesic therapy became mandatory, due to tumor-associated pain. Analgesics were titrated to 75 mcg/h fentanyl via a trans-dermal application system (SMAT) and oral metamizol (3 g/day) in combination with amitryptiline (25 mg/day), which led to sufficient pain control. Finally, a subclavian port device was implanted to facilitate supportive parenteral nutrition (1500 kcal/day) facing severe tumor-associated anorexia.

### Chemotherapy regime

Following informed consent, an experimental chemotherapy based on gemcitabine and anti-EGFR antibody (cetuximab) was initiated within the fourth week (25^th ^day) after first clinical presentation. Chemotherapy was administered via central venous catheter. The initiated schedule comprised applications of gemcitabine (1000 mg/m^2 ^bodysurface) every other week. The combination with cetuximab, starting with a loading dose of 400 mg/m^2 ^and followed by a weekly maintenance dose of 250 mg/m^2 ^bodysurface, was initiated together with the fourth gemcitabine cycle after financial approval for its experimental use. The patient received 20 cycles of chemotherapy for 42 weeks (i.e. 9.7 months) during the reported time period of 10.7 months since first presentation. The cumulative dose of gemcitabine reached 31.0 g/m^2 ^bodysurface and 14.9 g/m^2 ^bodysurface for cetuximab during the reported period.

### Chemotherapy-related toxicity and dose-modifications

Haematological toxicities were the most relevant gemcitabine-related side effects observed during chemotherapy, leading to severe leukopenia (CTC, grade 3), neutropenia (CTC, grade 2) and thrombocytopenia (CTC, grade 3). The haematological events required treatment with recombinant granulocyte colony-stimulating factor (filgrastim, 300 mcg/day) and transfusion of platelet concentrates, respectively. The patient was hospitalized to manage resulting neutropenic fever (CTC, grade 3) with intravenous antibiotics. Each documented episode of neutropenic fever was promoted by *Staphylococcus epiderdimis *sepsis, initiated by recurrent catheter-related infections. Thus, the revision of the infected port system abolished further recurrent catheter sepsis. Anorexia (CTC, grade 3), nausea (CTC, grade 3), alopecia (CTC, grade 1) and fatigue (CTC, grade 1) were other documented gemcitabine-related side effects. Toxicities caused by cetuximab were restricted to mild rosacea (CTC, grade 1) of the head and neck region. Other dermatological toxicities due to cetuximab did not develop during treatment. Severe complications under chemotherapy required hospitalization for 5.3 weeks (37 days), during which complete chemotherapy was paused. The second episode of chemotherapy-induced leukopenia has led to a prolonged dose reduction (50 percent) of gemcitabine for three subsequent cycles.

### Response to chemotherapy

**Figure 1:**
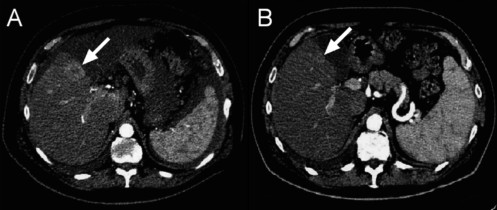
**Radiological follow up of hepatic tumor manifestations**. MS- CT axial: Representative nodule of the diffuse cholangiocarcinoma with marked arterial phase contrast enhancement (A), declining under chemotherapy (B).

**Figure 2:**
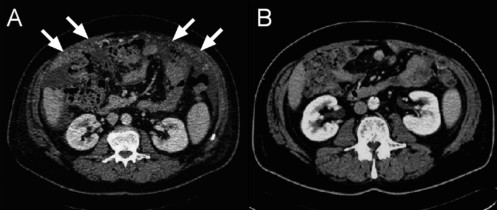
**Radiological follow up of peritoneal carcinomatosis**. MS- CT axial (contrast-enhanced, portal-venous phase): Peritoneal carcinomatosis (omental cake) (A), regressive under chemotherapy (B).

**Figure 3:**
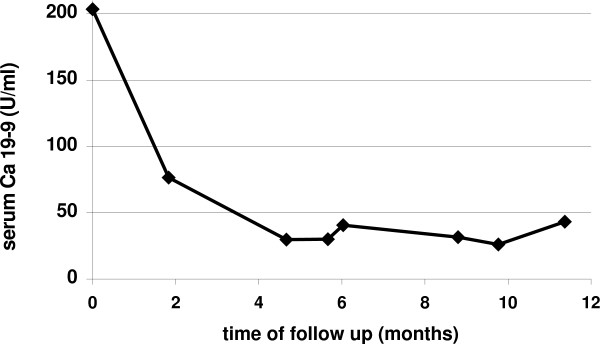
**Kinetics of Ca 19-9 concentrations during chemotherapy**. Quantification of Ca 19-9 serum concentrations by ELISA.

After nine cycles of chemotherapy abdominal CT-imaging showed a partial response, according to RECIST criteria. The hepatic target lesion showed a reduction in diameter of more than 30%. Additional hypervascularized non-target lesions, depicted in the hepatic arterial contrast phase, were no longer detectable after administration of the initial chemotherapy cycles. Peritoneal enhancement, representing peritoneal carcinomatosis, vanished completely after initiation of therapy. (Figure 1 and 2) Concordant with the radiological observations Ca 19-9 levels, which were elevated more than fivefold, returned to normal after 16 weeks of treatment. (Figure 3) During subsequent radiological follow up, tumor remission was persisting, showing ongoing stable disease for additional 26 weeks of treatment.

Most importantly, the clinical status (Karnofsky-Index: 70–80%) stabilized during chemotherapy and did not show any worsening until the end of follow up. Whereas the first three cycles of gemcitabine did not affect overall performance status significantly, tumor-associated anorexia improved after six cycles of combined gemcitabine and cetuximab. Therefore supplemental alimentation was discontinued, which was administered within the first four months after diagnosis. Also the initial analgesic dose of 75 mcg/h transdermal fentanyl could be reduced by 30% to 50 mcg/h.

## Conclusion

Expression of EGFR has been shown to be crucial for cancerogenesis in different tumor entities [9,10]. Moreover, EGFR-positive malignancies from bilio-digestive origin are associated with unfavourable outcome [11]. Therefore, EGFR-targeted therapies have been introduced to extended chemotherapeutic regimes. The fact that the addition of anti-EGFR antibodies to cytotoxic chemotherapy may rescue and increase response, suggests complementary anti-neoplastic mechanisms. The inhibition of EGFR homo- or hetero-dimerisation by EGFR antibodies interrupts cell signalling and initiates anti-neoplastic downstream effects, which lead to phosphorylation of tyrosine kinases. Phosphorylation of tyrosine kinases abrogates cell cycle progression and influences cellular adhesion, migration and differentiation [12,13]. In addition, angiogenesis is regulated by EGFR-dependant signal cascades and is modified by inhibitory anti-EGFR antibodies [14]. Despite cetuximab is not cytotoxic, subsequent apoptotic cell death may eventually lead to tumor involution.

Especially results from prospective trials addressing the response of colorectal cancer to cetuximab containing regimes have confirmed its efficacy in clinical practice. Second line therapies of colorectal cancer after failure of fist-line regimes show response rates of 17–23% and an overall survival (median) of 8.6 months [15,16]. Following these results, cetuximab has become the first monoclonal antibody for 2^nd ^line treatment of colorectal cancer, which has been approved by the US. Food and Drug Administration.

Even pancreatic adenocarcinoma, which is associated with unfavourable prognosis, is sensitive to cetuximab administration [6]. This has been shown by a phase II trial, comprising 41 patients with unresectable pancreatic adenocarcinoma. Patients in this trial required a confirmed EGFR expression and were treated with a fist-line therapy consisting of gemcitabine (1000 mg/m^2 ^weekly) and cetuximab (loading dose: 400 mg/m^2^, maintenance dose: 250 mg/m^2^). The promising results of this observation showed five patients (12.2%) who achieved a partial response and 26 (63.4%) with stable disease. The median time to disease progression was 3.8 months and the median overall survival duration was 7.1 months. One-year progression-free survival and overall survival rates were 12% and 31.7%, respectively.

Although the extent of EGFR expression of the target malignancy has not been associated with the overall response rate [17,18], we performed an immune-histochemical staining of the CC prior to treatment and were able to confirm a positive EGFR status. Subsequent follow up clearly showed a response by tumor reduction, which was maintained until end of follow up. The synergistic effect of cetuximab seems more convincing, if the reduced gemcitabine dosage is taken into account, which was administered every other week in contrast to a standardized weekly schedule (1000 mg/m^2 ^bodysurface). Even more pronounced dose reductions to only 37.4% of the standard dose were mandatory during the initial administrations of gemcitabine as single agent. Beside these promising results, cetuximab based palliative therapy was well tolerated. Even in combination with cytotoxic regimes, administration of cetuximab does not significantly increase frequency and severity of chemotherapy associated toxicity. Cutaneous toxicity is predominantly observed during cetuximab administration, while life threatening side effects are mainly restricted to cytotoxic drugs. In this reported case, only mild acneiforme rash could be addressed to cetuximab, whereas severe haematological and infectious complications were gemcitabine-related.

A significant reduction of analgesic medication and the discontinuation of parenteral nutrition have been the most striking clinical changes, after cetuximab has been added to the gemcitabine-based chemotherapy. As tumor-associated pain and tumor-associated anorexia influences individual performance status, the treatment significantly affected quality of life. Therefore, the combination of EGFR-specific antibodies with cytotoxic chemotherapy backbones, such as gemcitabine, is a promising combination for palliative treatment. Further prospective trials are mandatory to confirm the effects observed in this single case and to discriminate gemcitabine-induced from cetuximab-associated response.

## Competing interests

The author(s) declare that they have no competing interests.

## Authors' contributions

MFS and SK made substantial contributions to the acquisition, analysis and interpretation of the clinical data.

CCS, MM and PRG made substantial contributions to the concept and interpretation of the experimental treatment.

SSF contributed radiological follow up during this study.

All mentioned authors were substantially involved in critical reading of the manuscript and approved its final version.

## Pre-publication history

The pre-publication history for this paper can be accessed here:
